# A tale of two cascades: promoting a standardized tool for monitoring progress in HIV prevention

**DOI:** 10.1002/jia2.25498

**Published:** 2020-06-30

**Authors:** Judith D Auerbach, Annette AM Gerritsen, Gina Dallabetta, Michelle Morrison, Geoffrey P Garnett

**Affiliations:** ^1^ Department of Medicine University of California San Francisco San Francisco CA USA; ^2^ EpiResult Pietermaritzburg South Africa; ^3^ HIV Delivery TB/HIV Team Bill & Melinda Gates Foundation Washington DC USA; ^4^ HIV Epidemiology and Surveillance Bill & Melinda Gates Foundation Seattle WA USA; ^5^ Data and Metrics TB/HIV Team Bill & Melinda Gates Foundation Seattle WA USA; ^6^ Department of Global Health University of Washington Seattle Seattle WA USA

**Keywords:** HIV prevention cascades, HIV prevention programmes, prevention monitoring, programme improvement, key and vulnerable populations, public health, intervention

## Abstract

**Introduction:**

To achieve significant progress in global HIV prevention from 2020 onward, it is essential to ensure that appropriate programmes are being delivered with high quality and sufficient intensity and scale and then taken up by the people who most need and want them in order to have both individual and public health impact. Yet, currently, there is no standard way of assessing this. Available HIV prevention indicators do not provide a logical set of measures that combine to show reduction in HIV incidence and allow for comparison of success (or failure) of HIV prevention programmes and for monitoring progress in meeting global targets. To redress this, attention increasingly has turned to the prospects of devising an HIV prevention cascade, similar to the now‐standard HIV treatment cascade; but this has proven to be a controversial enterprise, chiefly due to the complexity of primary prevention.

**Discussion:**

We address a number of core issues attendant with devising prevention cascades, including: determining the population of interest and accounting for the variability and fluidity of HIV‐related risk within it; the fact that there are multiple HIV prevention methods, and many people are exposed to a package of them, rather than a single method; and choosing the final step (outcome) in the cascade. We propose two unifying models of prevention cascades‐one more appropriate for programme managers and monitors and the other for researchers and programme developers‐and note their relationship. We also provide some considerations related to cascade data quality and improvement.

**Conclusions:**

The HIV prevention field has been grappling for years with the idea of developing a standardised way to regularly assess progress and to monitor and improve programmes accordingly. The cascade provides the potential to do this, but it is complicated and highly nuanced. We believe the two models proposed here reflect emerging consensus among the range of stakeholders who have been engaging in this discussion and who are dedicated to achieving global HIV prevention goals by ensuring the most appropriate and effective programmes and methods are supported.

## 1 INTRODUCTION

The UN Political Declaration of 2016 outlined key *2020 Global Prevention Targets and Commitments* that included reducing the numbers of people newly infected with HIV to fewer than 500,000 per year (a 75% reduction against 2010 targets), reducing the number of adolescent girls and young women newly infected with HIV globally to below 100,000 per year, and ensuring that 90% of people at risk of HIV infection have access to comprehensive HIV prevention services. Notwithstanding a 16% reduction in HIV incidence overall between 2010 and 2018, it is clear that, as we have entered the year 2020, these global targets will not be met. To achieve significant progress from 2020 onward, it is essential to ensure that appropriate HIV prevention programmes are being delivered with high quality and sufficient intensity and scale and taken up by the people who most need and want them to have public health and epidemic impact.

Yet, currently, there is no standard way of assessing this. To redress this, attention increasingly has turned to the prospects of devising an HIV prevention cascade, similar to the now‐standard HIV treatment cascade; but this has proven to be a controversial enterprise, chiefly due to the complexity of primary prevention. We believe there is a way forward with two versions of a prevention cascade—a basic model that can be used by programme managers applying routine data, and an expanded model that can be used by researchers (working with programme managers) and public health policy makers who have access to and can generate additional types of data.

## 2 DISCUSSION

### 2.1 The value of prevention cascades

Prevention cascades have multiple, potential and somewhat overlapping uses. They can provide: a logical framework summarizing actions taken by individuals across a population that can prevent HIV acquisition; a management tool to focus on gaps and related barriers and bottlenecks and identify potential for programmatic improvement; and an advocacy tool for indicating points for intervention to enhance programme effectiveness.

### 2.2 Issues in devising a prevention cascade framework

Notwithstanding their potential value, a number of core issues and nuances have made it difficult to reach consensus in the field about how to devise HIV prevention cascades and to agree on one shared model. We address some of these below.

#### 2.2.1 *Presentation of* C*ascade*

Two different models of presenting the elements (or steps) in cascades have been used in the field, making comparisons in HIV treatment progress across locales difficult [1]. In one, the denominator remains the same for each step‐for example all people living with HIV; in the other, the denominator of each step is derivative from the step before—for example people living with HIV who know their status, of those, the proportion on ART, of those, the proportion who are virally suppressed [2]. The choice of model will, of course, influence its interpretation. Many in the HIV prevention field have been using “cascade” to signify models that show derivative denominators [3, 4, 5, 6, 7], as we do in the remainder of this commentary.

#### 2.2.2 The initial denominator

Determining the beginning denominator ‐ the population of interest ‐ in an HIV prevention cascade can be challenging. For HIV prevention, there is no uniform, clearly defined population in need (as compared with all people living with HIV for the treatment cascade), but several populations that have different characteristics and vulnerabilities to HIV infection. Moreover, HIV prevention is neither a uniform nor a linear process; people move in and out of situations of risk affected by a range of psycho‐social, interpersonal and demographic characteristics. This also means that within populations that are defined as “at risk” because of their overall HIV incidence or prevalence, for example female sex workers (FSW), there is heterogeneity of risk [8]. As with any group categorised according to epidemiological risk factors there is an averaging of risk across a heterogeneous population. In choosing a population focus there is a trade‐off in the level of risk and the number of people covered.

Although primary prevention cascades focus on those who are HIV negative, many HIV prevention programmes do not begin with HIV testing to determine who definitely is uninfected in order to focus the promotion of primary prevention methods on them. In some cases, prevention programmes are even intentionally delivered to a population that includes both HIV‐positive and HIV‐negative individuals with a specific type of risk for either transmission or acquisition, for example all people with non‐regular partners, for whom condoms would be recommended, which can complicate the denominator. With all these nuances in mind, size estimates of the population of interest provide the best, albeit imperfect method for determining the initial cascade denominator.

#### 2.2.3 Time covered by the cascade

Risk of HIV acquisition is cumulative over exposures occurring over time. The timeframe that the prevention cascade covers could vary depending on the intensity of risk and the duration prevention programmes are in place. In assessing HIV prevention programmes, it makes sense to track cohorts or to sample populations cross‐sectionally with time periods short enough to measure changes in appropriate use of interventions. In treatment cascades it is not always clear whether the 90:90:90 target refers to a specific moment in time, or is cumulative diagnosis, ART initiation and viral suppression for the cohorts of those infected. For prevention, the cascade should be assessed as a function of time; using convenient durations, such as a month or a year, as a standard timeframe will allow for comparability across prevention cascades.

#### 2.2.4 Multiple prevention options

People at risk of HIV often are presented with more than one option of prevention method, unlike the fairly singular ART option that is monitored through the treatment cascade [9]. Sometimes prevention method options are delivered simultaneously in a package, for example one that promotes condoms and pre‐exposure prophylaxis (PrEP) and provides economic support for adolescent girls and young women. Measuring the effect of a prevention package as a whole, while desirable, becomes tricky. The prevention package can be thought of as the activities undertaken to ensure that prevention methods are effectively used. Looking at the combined cascades generated by a prevention package allows the effectiveness of the package to be assessed. These realities of prevention programming mean that multiple, differentiated prevention cascades ‐ by population and prevention method ‐ are necessary. These can follow a uniform framework, but their characteristics and data points will be different. Fearon and colleagues [10] provide an example of a cascade, based on routine programme data, for each of two prevention methods provided to female sex workers ‐ condoms and PrEP ‐ showing where they overlap. This may be as close to assessing combination packages as we can come, outside of a large‐scale randomized community trial comparing the efficacy of different packages within similar populations, which, in fact, have not proven fruitful in the test and treat arena [11, 12, 13, 14].

#### 2.2.5 The final outcome

Deciding what exactly is and should be the ending point (the measured and reported outcome) for prevention cascades fundamentally depends upon the purpose of the cascade. If the focus is on programme coverage and the uptake and use of the prevention method(s) promoted by that programme, then the endpoint would be correct and consistent use (adherence or persistence) of the prevention method(s). If the focus is on evaluation or impact of the programme on HIV infection rates (incidence), then the endpoint would be remaining HIV negative. The latter is much more difficult to measure directly in a cascade model, as it requires a combination of programme data, behavioural surveys or interviews and HIV testing. But it can be based on estimates of efficacy when correct and consistent use is known. This is similar to how viral suppression ‐ not decreased morbidity, mortality, and/or onward transmission ‐ is used as the final outcome in the treatment cascade.

Because cascades measure the reduction in risk across individuals but do not account for reduced exposures as the prevalence of infection falls, they provide a partial population level measure of the impact of prevention interventions. Where capacity exists, researchers can work with programme implementers to estimate infections averted through mathematical modelling of the acquisition and transmission of HIV and the population dynamics of infections and other extrapolation methods.

### 2.3 Two unifying cascade models

While the issues noted above continue to be debated, a few consensus meetings have reached the conclusion that HIV prevention programme managers at national and sub‐national levels and from government and non‐governmental organisations who are involved in the implementation, administration, monitoring and evaluation of HIV prevention programmes would benefit from having a standardised cascade model, like the treatment cascade, to monitor their progress and identify gaps and opportunities for improvement [15]. The advantage of a relatively simple, generic model is that it promotes comparability over time, across populations, between sub‐groups of a population, across geographical areas and between prevention methods. Moreover, it can be populated with routine programme data and survey data.

**Figure 1:**
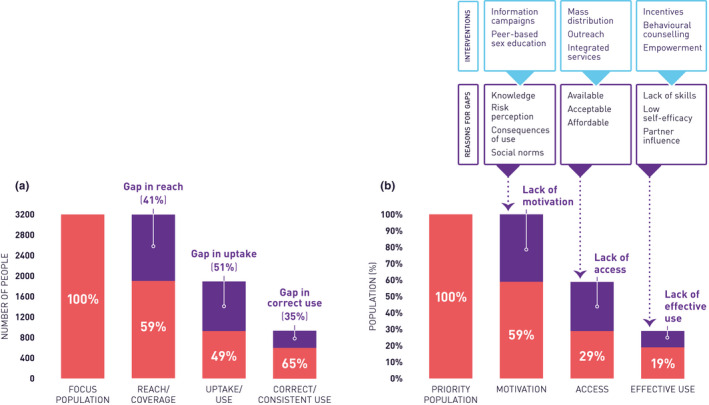
Two unifying prevention cascade models. In the basic model **(a)**, the bars represent the programme success, showing, sequentially, the number and proportion of the focus population that is covered by the intervention (59%), the number and proportion of those covered by the intervention that take it up (49%) and the number and proportion of those who take up the intervention that use it correctly (65%) (e.g. in the past 12 months). The purple area and proportion above each red section of the bar represents the gap in each step. This cascade is recommended for assessing basic programme performance. In the expanded model **(b)**, using the same data, but a format adapted from Schaefer and colleagues [5] and Manicaland Centre [15] (whereby the percentages across the red bars are based upon the priority population), the gaps are in motivation, access and consistent use, and the reasons for these can be further explored and potential interventions identified. This alternative approach is more attuned to research and the design of programmes than to the monitoring of programme implementation.

A collaboration of global partners, including the authors and representatives from UNAIDS, WHO, and a number of national HIV prevention programmes, are developing operational guidance for creating basic HIV prevention cascades that can be used as a programme management tool [16] that can describe what is being provided and used by a given population and determine where the gaps lie for the purposes of informing programme improvement, as shown in Figure 1 (model a). This basic cascade model begins with identification and quantification of a population group that is the focus of a prevention programme. Ideally, this population will be comprised of those most at risk of getting infected as well as most in need of the particular prevention method(s) being provided by the programme, although, as noted above, this may not always be the case. It can best be determined using size estimation methods, for example census, enumeration, behavioural surveillance, mapping, etc. [17] The next step, reach or coverage, is defined as the extent to which a prevention method is delivered or made accessible to the focus population. The third step measures uptake and initial use of the prevention method among those reached by the programme; and the fourth step assesses the extent to which those who take up a prevention method use it correctly and consistently. Reach, uptake and use may be measured with routine programme data, polling booth survey data, and, if feasible, behavioural surveillance data. This basic model has been adopted for prevention programme monitoring in Kenya [18, 19], India [20] and Zimbabwe [21].

The basic prevention cascade, effectively, is the first step in a three‐step framework that would subsequently involve qualitative and quantitative research to understand the individual and social‐structural causes (i.e. the why) of these gaps, and then the development and testing of appropriate solutions to improve the effectiveness of HIV prevention efforts, as demonstrated ultimately by reductions in HIV incidence, although that may not be represented in the cascade itself. A version of this extended framework, modified from that proposed by Schaefer and colleagues [5] and informed by earlier work [3, 4, 15], is also shown in Figure 1 (model b). It includes a step related to “motivation” to denote the importance of “demand” as a facilitator in uptake and use of a prevention method. This model, recently applied in Manicaland, Zimbabwe [22], is most feasible to use in contexts with research capacity and infrastructure, as it requires collection and analysis of quantitative and qualitative data that go beyond the data regularly collected by prevention programme implementers and managers. It is worth noting that in some circumstances, the order of the motivation and access steps in the Schaefer et al. model would differ. For example when everyone in a focus population has access to a prevention method, “motivation” might come after “access” to better convey who in the end actually takes up the prevention method because they want to use it when availability is not an obstacle.

Furthermore, in Figure 1 model b, the proportion shown in the final step (effectively using the method) represents the overall success of the programme vis‐à‐vis the total focus population. This value could be multiplied by known efficacy of the prevention method(s) promoted in the programme to estimate the proportional reduction in HIV incidence achieved. The difference in the two methods of cascade illustration is important for the immediate visual impression it makes on how well a programme is performing, but the information on the scale and relative importance of gaps is similar.

### 2.4 Data quality and improvement

A prevention cascade will only be as good as the data that comprise it. Since most data are not perfect, neither will be the prevention cascade. What is most important is to use the best available data, while continuing to find ways to improve them, and to use these data to inform decisions about programme improvement. When engaging in cascade analysis, it is important to be clear about the sources, quality and limitations of the data used, as each has its strengths and limitations, and each can be improved [23, 24, 25].

For example when no valid size estimates are available for members of a key population group who are most at risk, multiple estimates may have to be generated and triangulated to reach both a point estimate and a range. Integrated bio‐behavioural surveillance (IBBS), while employing a consistent sampling method and ensuring representativeness, still relies on self‐report, which is subject to bias. Similarly, polling booth and small areas surveys, which are more easily used in programmes also may be subject to sampling bias. This can be improved by introducing innovative, and potentially more valid, data collection methods, such as digital methods. Additionally, programmes may not routinely collect all the data needed to populate a cascade, nor disaggregate them by key population. But these data can be triangulated with other sources, including IBBS and focused qualitative surveys, where feasible. In sum, efforts should be made to assure that the highest quality data available are used in cascade development and analysis, and, simultaneously, to improve upon their quality in new and innovative ways.

## 3 CONCLUSIONS

The HIV prevention field has been grappling for years with the idea of developing a standardised way to regularly assess progress and to monitor and improve programmes accordingly. The cascade framework, already universally adopted for HIV treatment, provides the potential to do this, but it is complicated and highly nuanced. We have proposed that two models be employed—one chiefly for programme managers, and the other chiefly for programme developers, researchers and policy makers—to move the field forward. We believe these models reflect emerging consensus among the range of stakeholders who have been engaging in this discussion and who are dedicated to achieving global HIV prevention goals by ensuring the most appropriate and effective programmes and methods are supported.

## COMPETING INTERESTS

J.D.A: none, A.A.M.G.: none, G.D.: none, M.M.: none, G.P.G.: none.

## AUTHORS’ CONTRIBUTIONS

J.D.A., A.A.M.G., G.D., M.M. and G.P.G. conceived the idea for this commentary: J.D.A. and A.A.M.G. prepared the manuscript. All authors reviewed, provided feedback and approved the final manuscript.
